# Spatial role of land cover on West Nile virus disease in Europe

**DOI:** 10.1016/j.isci.2026.115754

**Published:** 2026-04-15

**Authors:** Nicola Riccetti, Alessandro Cescatti, Juan Carlos Ciscar, Grégoire Dubois, Angela Fanelli, Jordi Figuerola, Dolores Ibarreta, Wojciech Szewczyk, Emanuele Massaro

**Affiliations:** 1 European Commission, Joint Research Centre (JRC), Ispra, Italy; 2 European Commission, Joint Research Centre (JRC), Sevilla, Spain; 3 Estacion Biologica de Doñana, Consejo Superior de Investigaciones Científicas (CSIC), Avda. Americo Vespucio 26, 41092 Sevilla, Spain; 4 Centro de Investigación Biomédica en Red Epidemiología y Salud Pública (CIBER ESP), Avda. Monforte de Lemos, 3-5, 28029 Madrid, Spain

**Keywords:** Disease, Microbiology, Viral microbiology

## Abstract

We analyzed West Nile virus (WNV) disease incidence across European provinces from 2005 to 2019. Using spatial regression models, we quantified how land-cover gradients, climatic conditions, and socio-demographic variables jointly shape spatial heterogeneity in WNV disease incidence in humans. Shrubland cover showed the strongest and most spatially consistent positive association with human WNV disease incidence, whereas forest cover generally exhibited a negative relationship; urban and cropland areas had weaker, regionally variable effects. Climatic factors—particularly warm summer temperatures and seasonal moisture balance—emerged as dominant predictors, while socio-economic variables contributed little at this scale. The spatially adaptive spatially lagged geographically weighted regression (GWR-SL) model revealed pronounced regional variation in these associations, highlighting that WNV disease drivers are highly context dependent. These findings underscore the value of integrating land-cover and climatic information into targeted surveillance and vector-control strategies, while acknowledging limitations related to land-cover aggregation, climatic averaging, and underreporting.

## Highlights

• Shrubland cover has the strongest positive link to West Nile virus incidence in Europe
• Warm summer temperatures and moisture balance are the dominant climatic predictors
• The spatial model shows that disease drivers are highly dependent on regional context
• Targeted surveillance should prioritize shrub-dominated and warm peri-urban landscapes

## Graphical abstract

**Figure:**
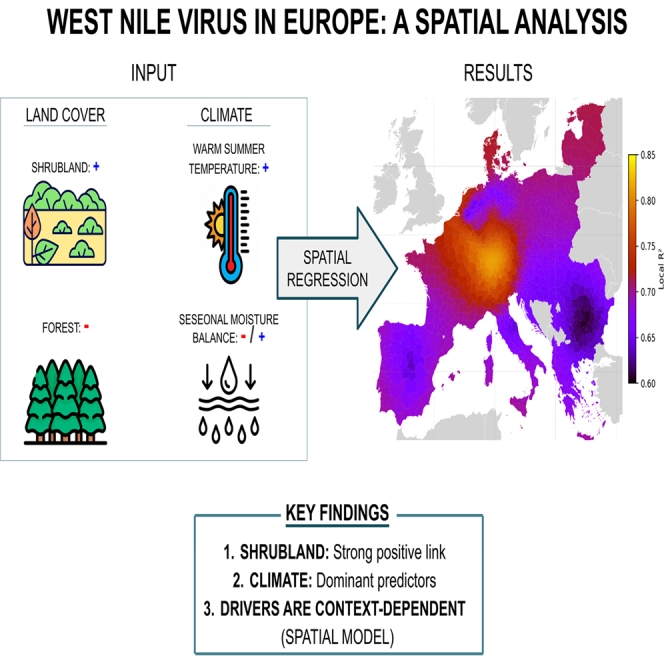

## Introduction

Although West Nile virus (WNV) disease generally causes relatively low mortality in humans,^1^^,^^2^^,^^3^^,^^4^ it represents one of the most widespread mosquito-borne viral infections in the world. WNV is maintained in a zoonotic cycle involving Culex mosquitoes—particularly *Culex pipiens* and *Cx. modestus* in Europe—and avian hosts belonging mainly to the order *Passeriformes* and particularly to the genus *Corvus*. Humans and other mammals are incidental, dead-end hosts who become infected through mosquito bites but do not contribute to further transmission.

Since its introduction and subsequent establishment in Europe, WNV has expanded geographically and re-emerged periodically in several regions, causing seasonal outbreaks that vary in magnitude and spatial distribution.^5^^,^^6^^,^^7^^,^^8^^,^^9^^,^^10^ This resurgence has raised growing concern among public-health authorities, particularly under changing climatic and environmental conditions that may increase viral circulation in the future.^10^ No licensed vaccine for the disease or specific antiviral treatment for WNV is currently available.^11^ Consequently, public-health strategies rely primarily on reducing mosquito-human contact through vector control and personal protection measures, and on improving surveillance and early-warning systems.

Predictive models have played a crucial role in guiding these preventive efforts. Historically, most studies of mosquito-borne viruses have emphasized climatic and environmental factors—such as temperature, precipitation, humidity, vegetation, surface water, and land use/cover—because these variables directly and indirectly influence mosquito abundance, development, habitat suitability, and viral replication.^12^^,^^13^^,^^14^^,^^15^ However, temperature effects on mosquito populations and virus transmission are often non-linear.

In fact, several mosquito life traits show a unimodal relationship with temperature (e.g., larval survival, development rate, and biting frequency), whereas adult lifespan tends to decrease beyond approximately 30°C, indicating that excessively high temperatures can reduce vector survival.^16^ Excessive temperatures can therefore suppress vector populations, both directly through reduced survival and indirectly through decreased fecundity and altered blood-feeding behavior.^9^^,^^17^^,^^18^ Similarly, vector competence and viral transmission efficiency peak at intermediate temperatures (typically 30°C–36°C, depending on the vector-pathogen combination).^19^

Because of these complex and context-dependent relationships, a growing number of studies have called for more integrative frameworks that incorporate not only climatic drivers but also land-cover characteristics and socio-demographic factors.^9^ Land use and anthropogenic environmental change can reshape mosquito and host habitats,^17^ alter the composition and abundance of competent vectors, and modulate exposure risk for human populations.

In this context, our study focuses specifically on WNV disease incidence across European provinces from 2005 to 2019. We examine how land-cover types—particularly forests, croplands, shrublands, and urban areas—interact with climate and socio-demographic variables to explain spatial heterogeneity in WNV disease incidence in humans. By quantifying how the relative importance of these factors varies across Europe, we aim to provide insights that can inform public-health planning and spatially targeted surveillance for this zoonotic disease.

## Results

To investigate the spatial determinants of WNV disease incidence in humans, we included only cases with a known province of infection. A total of 7,909 WNV disease cases in humans were recorded during the study period, of which 3,517 with a known province of infection (44%). After filtering cases with missing values in other covariates of the model, we finally included in our model 3,379 WNV disease cases in humans (43%).

### Descriptive results

**Figure 1:**
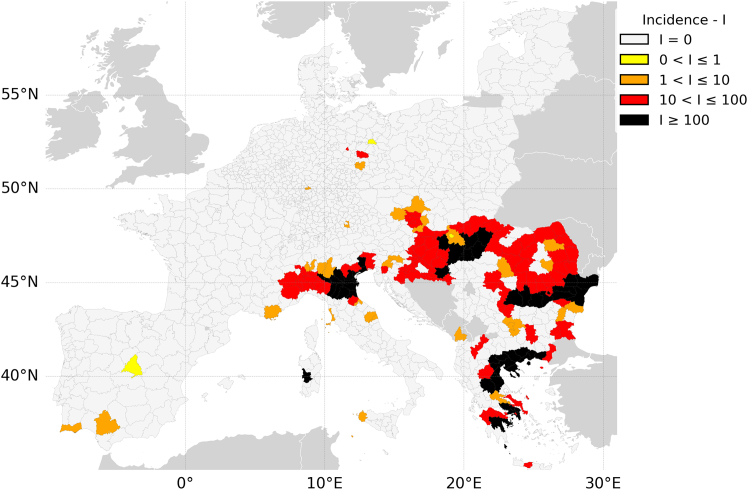
Incidence of West Nile virus disease in Europe Incidence (I) of West Nile virus disease, expressed as human cases per 100,000 inhabitants, in continental Europe from 2005 to 2019, at the NUTS 3 level (nomenclature of territorial units for statistics). The analysis includes only cases where the province of infection was reported. The legend illustrates the five value classes used in the plot.

In the time frame and area of our analysis, the provinces accounting for the most cases were the Northern Greek province of Thessaloniki (160 cases), the Romanian province of Bucharest (138 cases), and the Italian province of Padua (120 cases). The provinces with the highest incidence/100,000 inhabitants were again all in Northern Greece: Xanthi (1,384 cases/100,000), Imathia (1,239 cases/100,000), Pella (1,219 cases/100,000), and Thasos, Kavala (929 cases/100,000) (Figure 1).

### Regression analysis

**Figure 2:**
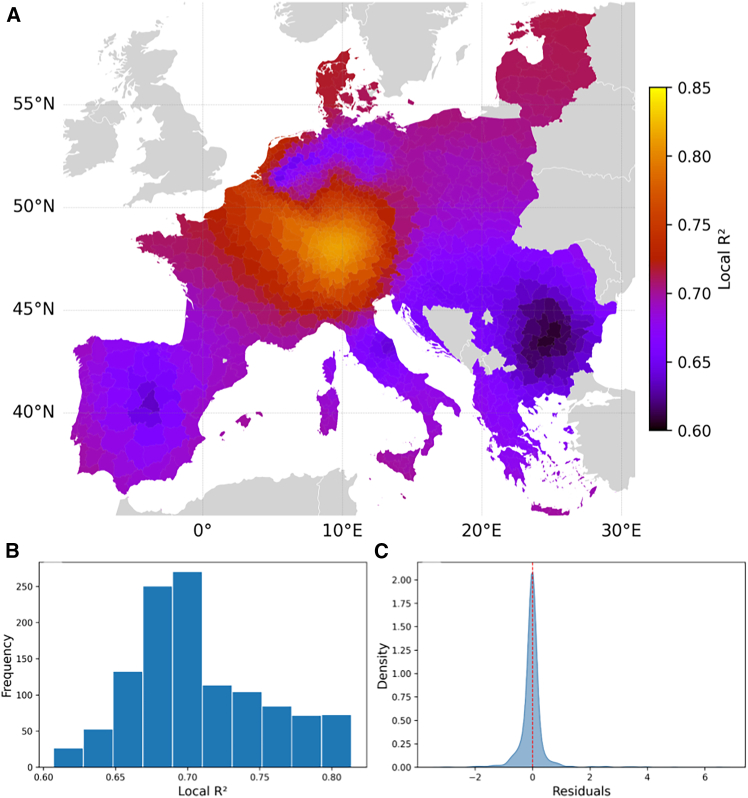
Descriptive statistics of the covariates potentially associated factors (A) Distribution of the different land cover variable throughout the entire area of interest (up). (B) spatial distribution of main land cover variable per each NUTS3 level. (C) Spatial distribution of population density and (D) gross-domestic product per person (GDP per capita).

The global Ordinary Least Squares (OLS) model was statistically significant (F(15, 1156) = 58.1, *p* < 0.001) and explained 46% of the variance in the spatially lagged WNV disease incidence in humans (R^2^ = 0.466, adjusted R^2^ = 0.458; Table 1). Among standardized predictors, the strongest effects were observed for mean spring temperature (β = −2.72, *p* < 0.001), mean summer temperature (β = 4.11, *p* < 0.001), and maximum number of consecutive dry days in spring (β = −0.53, *p* < 0.001). Because all variables were standardized, coefficients are expressed in standard deviation (SD) units of spatially lagged WNV disease incidence per one-SD change in the predictor. The spatially lagged geographically weighted regression (GWR-SL) model improved fit considerably (corrected Akaike information criterion [AICc] = 2089.2; global R^2^ = 0.72; adjusted R^2^ = 0.69). Local R^2^ values ranged from 0.47 to 0.81 (2.5^th^–97.5^th^ percentile range = 0.55–0.78; mean = 0.69; median = 0.70; Figure 2). This spatial variation in model performance indicates that the strength of environmental and socio-economic effects on WNV disease incidence varies regionally across Europe.

**Table 1:** Summary of global (linear model, LM) and local (geographically weighted regression model, GWR) regression results

Covariate	LM Estimate	LM SE	LM *p* value	GWR mean	GWR SD	GWR Min	GWR Median	GWR Max
(Intercept)	0	0.021	1	−0.095	0.074	−0.207	−0.108	0.041
Forest	−0.068	0.03	0.021	−0.034	0.035	−0.121	−0.031	0.028
Shrub	0.196	0.025	0	0.126	0.184	−0.206	0.076	0.47
Urban	−0.072	0.027	0.007	−0.014	0.048	−0.159	−0.002	0.082
Crop	−0.116	0.032	0	−0.075	0.151	−0.77	−0.02	0.065
Water	0.04	0.024	0.098	0.026	0.026	−0.002	0.018	0.147
Other	0.003	0.026	0.903	−0.01	0.021	−0.141	−0.006	0.046
Mean_P_Winter	−0.136	0.045	0.002	0.009	0.141	−0.563	0.063	0.152
Max_WD_Winter	−0.054	0.049	0.268	−0.09	0.096	−0.503	−0.068	0.036
Max_DD_Winter	−0.365	0.07	0	0.002	0.326	−1.078	0.142	0.379
Mean_T_Spring	−2.718	0.259	0	−1.274	1.187	−4.405	−0.728	−0.183
Max_DD_Spring	−0.532	0.042	0	−0.316	0.257	−1.225	−0.217	−0.066
Mean_T_Summer	4.112	0.295	0	1.945	1.979	0.253	1.107	7.836
Max_WD_Autumn	0.016	0.041	0.689	−0.023	0.086	−0.229	−0.018	0.377
Max_DD_Autumn	0.025	0.071	0.724	0.014	0.068	−0.318	0.032	0.105
gdp	−0.041	0.027	0.138	−0.021	0.018	−0.085	−0.018	0.026
pop_dens	0.034	0.025	0.174	−0.004	0.02	−0.087	−0.003	0.062

Metric	LM	GWR
Residual SE (df)	0.736 (1157)	0.558 (1066.3)
Residual SS	627.364	332.156
Multiple R^2^	0.466	0.717
Adjusted R^2^	0.458	0.688
F-statistic (df)	63.1 (16; 1157)	
F *p* value	<0.001	
AICc	2632.579	2089.216
Local R^2^ P2.5		0.55
Local R^2^ P97.5		0.78
Local R^2^ Mean		0.69
Local R^2^ Median		0.7

For the LM, we report standardized coefficients, standard errors, *p* values, and model diagnostics including residual standard error (with degrees of freedom), Multiple R^2^, Adjusted R^2^, F-statistic (with degrees of freedom), and *p* value. For the GWR, we report the mean, standard deviation, and range (minimum, median, maximum) of local parameter estimates and the 5^th^ (P5), 95^th^ (P95), mean, and median values of the local R^2^. All models were estimated using standardized variables (predictors globally *Z* scored; dependent variable standardized after spatial lagging at 150 km).

### Land use factors

**Figure 3:**
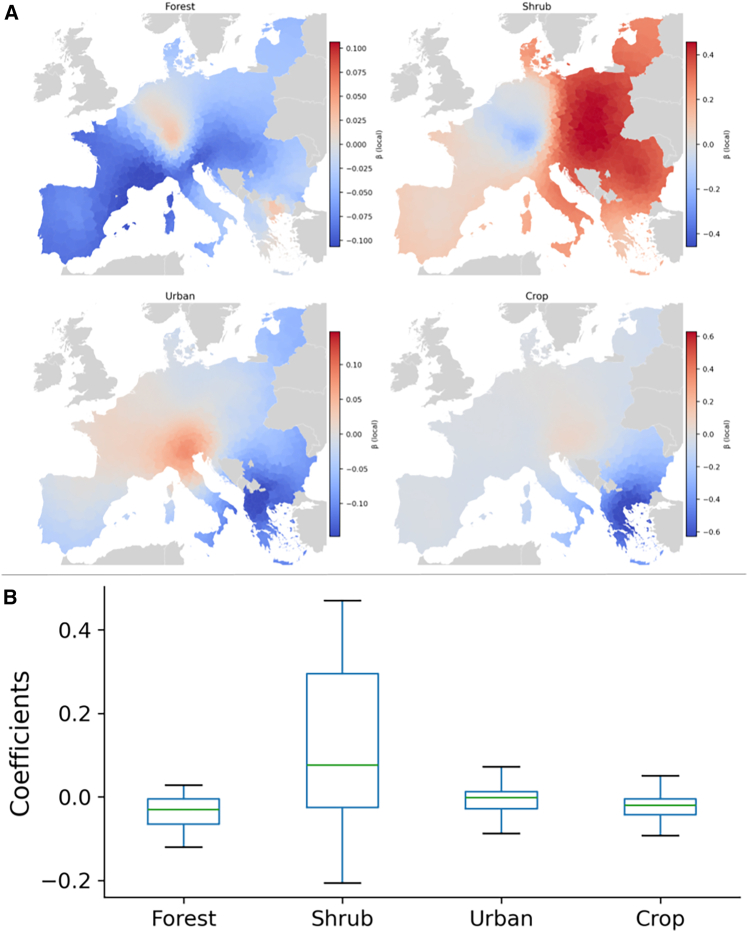
Spatial variation in the effects of land-cover variables on WNV disease incidence in humans across Europe (A) Local coefficients estimated by the geographically weighted regression (GWR) model for the four significant land-cover predictors: forest, shrub, urban, and cropland cover. Warm colors indicate positive associations with WNV disease incidence in humans and cool colors indicate negative associations. Each map uses a variable-specific symmetric color scale. (B) Distribution of local coefficients for the same predictors, shown as boxplots summarizing their spatial variability (median, interquartile range, and 5^th^–95^th^ percentiles). All predictors were standardized prior to modeling, so coefficients represent standardized effects (in SD units) on province-level WNV disease incidence in humans.

Spatial patterns in the influence of land-cover variables revealed clear geographic contrasts across Europe (Figures 3A and 3B). Shrubland cover showed the strongest and most spatially consistent positive association with WNV disease incidence, particularly across central and eastern Europe, where local coefficients reached their highest values. Provinces in Germany, Austria, and parts of Eastern Europe exhibited positive relationships, suggesting that semi-natural shrub-dominated landscapes may favor mosquito habitats or reservoir host presence. In contrast, forest cover was negatively associated with WNV disease incidence in humans throughout most of Europe, with the strongest negative coefficients in southern and western regions, such as Italy, France, and the Iberian Peninsula. Urban and cropland areas displayed weaker and more heterogeneous effects. Urban cover tended to show slightly positive coefficients in parts of Central Europe but negative associations in southern regions, indicating that urbanization may not uniformly enhance WNV disease risk in humans. Cropland showed mostly weak negative effects, with stronger decreases in Southeastern Europe, where intensive agricultural land use may reduce suitable breeding or reservoir conditions. Overall, land-cover predictors together highlight a gradient from semi-natural landscapes associated with higher WNV disease incidence in humans to more forested or intensively cultivated areas showing a suppressive effect.

**Figure 4:**
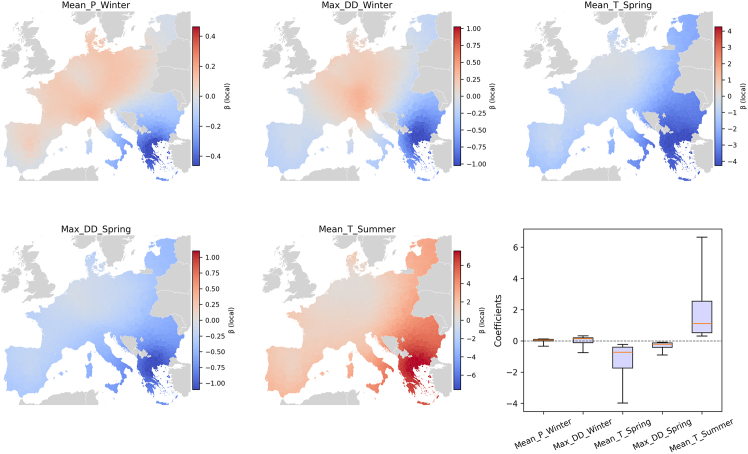
Spatial variation in the effects of climate variables on WNV incidence across Europe Local coefficients estimated by the geographically weighted regression (GWR) model for the five significant climate predictors: mean winter precipitation (Mean_P_Winter), maximum number of consecutive dry days in winter (Max_DD_Winter), mean spring temperature (Mean_T_Spring), maximum number of consecutive dry days in spring (Max_DD_Spring), and mean summer temperature (Mean_T_Summer). Warm colors indicate positive associations with WNV disease incidence in humans, and cool colors indicate negative associations. Each map uses a variable-specific symmetric color scale. The boxplot summarizes the distribution of local coefficients for all predictors (median, interquartile range, and 5^th^–95^th^ percentiles). All variables were standardized prior to modeling, so coefficients represent standardized effects (in SD units) on province-level WNV disease incidence in humans.

Climatic predictors exhibited strong and spatially structured effects on WNV disease incidence in humans (Figure 4). Mean summer temperature showed the most pronounced positive relationship, particularly across Southeastern and Eastern Europe, with the highest coefficients observed in Greece, Bulgaria, and Romania, indicating that warmer summer conditions substantially increase transmission risk. Mean spring temperature displayed a consistent negative association over much of Europe, especially in Southern regions, suggesting that areas experiencing unusually warm springs may experience earlier but less intense WNV disease human incidence amplification later in the season. The number of consecutive dry days in spring (Max_DD_Spring) also tended to correlate negatively with incidence, with stronger effects in Southern and Eastern provinces, reflecting the importance of moisture availability for vector breeding. In contrast, both mean winter precipitation and maximum consecutive dry days in winter (Max_DD_Winter) showed weaker and spatially mixed effects, with slight positive associations in Western and Central Europe and negative ones in the Southeast. Overall, these spatial patterns highlight the dominant role of warm summer conditions and seasonal moisture balance in shaping regional WNV disease dynamics in humans across Europe.

## Discussion

Using a GWR-SL model at the provincial level across continental Europe (2005–2019), we aimed to disentangle the local influence of land cover and climatic factors on the incidence of WNV disease in humans. Emerging and re-emerging mosquito-borne diseases (MBDs) remain a growing public health challenge, exposing millions of individuals to infection risk each year.^5^^,^^6^^,^^7^ Control strategies primarily focus on reducing contact between humans and mosquitoes, both through personal protection and targeted vector control. These interventions are often guided by epidemiological models designed to identify the most suitable timing and location for preventive measures to be implemented.^12^ While climatic variables such as temperature and precipitation are well-established determinants of MBDs’ dynamics,^13^^,^^14^^,^^15^^,^^20^ an increasing body of research shows that broader environmental factors—including land use, vegetation, and surface water—also play a major role in shaping WNV disease risk.^12^ Our analysis builds on this understanding by explicitly quantifying the joint influence of land-cover and climatic gradients on human WNV disease incidence at a fine spatial resolution across Europe.

Due to the availability of consistent data, the analysis focused on WNV disease, the only viral MBD with sufficient long-term coverage at the provincial scale. Our results show that several land-cover and climatic predictors were significantly associated with WNV disease incidence in humans, whereas socio-economic variables (GDP per capita and population density) did not show any significant association with WNV disease incidence. This finding suggests that, at this spatial scale, environmental heterogeneity—rather than economic or demographic differences—explains most of the spatial variation in disease incidence.

### Land-cover effects

Among land-cover variables, shrubland cover showed the strongest and most spatially consistent positive association with WNV disease incidence in humans, particularly across Central and Eastern Europe. These semi-natural environments may provide favourable conditions for *Culex* mosquitoes and avian reservoirs by combining vegetation density with suitable microclimatic conditions and standing water availability. In contrast, forest cover exhibited a significant negative association, especially in Southern and Western Europe, suggesting that closed-canopy forests may reduce vector breeding or host-vector contact. Urban and cropland cover had weaker, spatially variable effects. Urban areas tended to show small negative associations overall, with localized positive values in Central Europe, which could be consistent with the previously suggest adaptability of *C. pipiens* to peri-urban environments.^12^^,^^17^^,^^19^^,^^21^ Cropland, especially in intensively cultivated regions of Southeastern Europe, showed predominantly negative relationships, possibly reflecting the lower persistence of breeding sites in managed agricultural systems. Collectively, these patterns indicate that land-cover configuration—the balance between natural, semi-natural, and managed habitats—strongly mediates local WNV disease incidence risk and should be considered alongside climate in predictive and management frameworks.

### Climatic effects

Climatic variables exhibited strong and spatially structured associations with WNV disease incidence in humans. Mean summer temperature was the most influential predictor, showing a pronounced positive effect across Southeastern and Eastern Europe, consistent with the known role of high temperatures in accelerating mosquito development, viral replication, and transmission efficiency.^20^ Conversely, mean spring temperature showed negative associations across Southern Europe, suggesting that early-season warming may advance mosquito activity but shorten the window for peak viral amplification. The number of consecutive dry days in spring also correlated negatively with WNV disease incidence in humans, especially in Southern and Eastern regions, underscoring the role of surface moisture in sustaining mosquito habitats. Winter conditions (precipitation and dry days) displayed weaker and spatially mixed effects, with modest positive associations in Central and Western Europe. Together, these findings confirm that seasonal temperature and moisture balance are central determinants of WNV transmission suitability across the continent.

It is important to note that these climatic coefficients describe long-term suitability patterns rather than short-term fluctuations. Because seasonal means were averaged over 15 years, the model reflects spatial differences in baseline climatic favourability rather than temporal responses to specific extreme events or interannual anomalies.

### Model performance and spatial heterogeneity

The GWR-SL model substantially improved model fit compared to the global OLS regression. While the OLS model explained approximately 46% of the variance in WNV disease incidence in humans, the local GWR-SL model achieved a mean local R^2^ of 0.69, ranging from 0.47 to 0.81. This improvement highlights that relationships between WNV disease incidence in humans and environmental factors vary significantly across space, reinforcing the need for geographically explicit models. Spatially adaptive approaches like GWR-SL allow for the identification of regional clusters where specific variables—such as shrubland cover or summer temperature—exert stronger effects, providing a nuanced understanding of ecological risk gradients.

### Limitations of the study

Some limitations must be acknowledged. The aggregation of detailed land-cover types into broad categories (e.g., “cropland” or “forest”) may obscure finer ecological variations, such as irrigation or vegetation structure, that influence mosquito habitats. In this context, further research on the topic might be looking at different land cover groupings or more granular land cover classes, both in single terms as well as in their interactions. Similarly, the aggregation of the climatic variables in long-term seasonal averages represents rather a broad climatic suitability rather than climatic variability or extreme events; also in this context, further research could aim at disentangling the role of climatic variables in WNV disease incidence in humans, within a geographically weighted regression approach. Differences in *Culex* vector species and avian host distributions across Europe could further explain regional contrasts in model coefficients. Underreporting also remains a concern, given that up to 80% of human WNV diseases are asymptomatic,^18^ potentially biasing incidence data toward countries with stronger surveillance systems. This becomes even more relevant when considering that our approach allowed us to consider only WNV disease cases in human, which had a non-missing province of infection. This criterion—alongside the necessity to exclude from the model records with missing values in at least one of the covariates—has inevitably reduced our study population from 7,909 to 3,379 (43%) human cases and may have introduced additional bias.

Despite these limitations, the findings offer clear implications for public health and environmental management. Rather than prescribing direct land-cover modification (e.g., removing shrubs or draining wetlands), the results highlight where specific combinations of land and climate conditions create favourable ecological niches for WNV transmission. These areas—particularly shrub-dominated or peri-urban landscapes under warm summer regimes—should be prioritized for vector surveillance, larval habitat monitoring, and community awareness programs. In practice, such evidence can inform targeted vector control (e.g., larvicide in semi-natural zones), urban planning measures (e.g., maintaining vegetative buffers away from standing water), and risk communication in high-risk regions. Conversely, recognizing areas where forested or intensively cultivated landscapes act as natural buffers may help optimize the allocation of limited surveillance and control resources.

Overall, this study underscores that understanding the spatial interplay between land use and climate is essential to anticipate WNV disease risk under current and future environmental change. As Europe faces ongoing warming and land-use intensification, integrating ecological indicators into early warning systems will be vital to improve outbreak preparedness, guide localized interventions, and reduce the public health burden of mosquito-borne diseases.

## Resource availability

### Lead contact

Requests for further information and resources should be directed to and will be fulfilled by the lead contact Nicola Riccetti, nicolariccetti@gmail.com.

### Materials availability

This study did not generate new unique reagents.

### Data and code availability

• All data reported in this paper will be shared by the lead contact upon request.
• All original code used for this work has been deposited at GitHub and is publicly available as of the date of publication. https://github.com/emanuelemassaro/WNV_Europe/
• Any additional information required to reanalyze the data reported in this paper is available from the lead contact upon request.

## Acknowledgments

The study was supported by the Exploratory Project APES of the 10.13039/501100000780European Commission, 10.13039/501100000900Joint Research Centre. J.F. would like to thank ARBOPREVENT from 10.13039/100010434Fundación 'la Caixa' that partially supported this work.

## Author contributions

Conceptualization, N.R. and E.M.; revision data analysis, E.M.; data curation and formal analysis, N.R. and E.M.; funding acquisition, N.A.; investigation, methodology, project administration, resources, and software, N.R. and E.M.; supervision and validation, E.M.; visualization and writing – original draft preparation, N.R.; final paper preparation and revision, E.M.; writing – review and editing, N.R., A.F., W.S., A.C., J.C.C., G.D., D.I., J.F., and E.M.

## Declaration of interests

The authors declared no competing interests.

## STAR★Methods

### Key resources table

**Table:** 

REAGENT or RESOURCE	SOURCE	IDENTIFIER
**Deposited data**		

Mosquito-borne diseases human case data (Chikungunya, Dengue, West Nile virus, Yellow Fever, Zika)	European Center for Diseases Prevention and Control (ECDC)^22^	https://www.ecdc.europa.eu/en/publications-data/access-eueea-surveillance-data-third-parties
Land cover raster files	European Space Agency (ESA)^23^	https://climate.esa.int/en/#/
Climatic data (ERA5)	European Center for Medium-Range Weather Forecasts (ECMWF)^24^	https://cds.climate.copernicus.eu/datasets/reanalysis-era5-single-levels?tab=overview
Socio-demographic data	European Statistical Office (EUROSTAT)^25^	https://ec.europa.eu/eurostat/data/database

**Software and algorithms**		

Python (v3.14.0)	Python Software Foundation^26^	https://www.python.org/downloads/release/python-3140/
libpysal (PySAL core library v4.13.	Rey, S. J. et al.^27^	https://asu.elsevierpure.com/en/publications/pysal-a-python-library-of-spatial-analytical-methods/
spreg (Spatial Regression v1.8.4)	Rey, S. J., & Anselin, L.^27^	https://asu.elsevierpure.com/en/publications/pysal-a-python-library-of-spatial-analytical-methods/
Statsmodels (v0.14.5)	Seabold, S., & Perktold, J.^28^	https://doi.org/10.25080/Majora-92bf1922-011
MGWR (Multiscale GWR v2.2.1)	Oshan, T. M et al.^29^	https://www.mdpi.com/2220-9964/8/6/269
scikit-learn (v1.7.2)	Pedregosa, F. et al.^30^	https://jmlr.org/papers/volume12/pedregosa11a/pedregosa11a.pdf
GeoPandas	Jordahl, K. et al.^31^	https://doi.org/10.5281/zenodo.15750510
Shapely (v2.1.2)	Gillies, S. et al.^32^	https://doi.org/10.5281/zenodo.7428463
Rasterio	Gillies, S. et al.^33^	https://sgillies.net/2013/11/24/introducing-rasterio.html
NumPy (v2.3.5)	Harris, C. R. et al.^34^	https://www.nature.com/articles/s41586-020-2649-2
pandas (v2.3.3)	Pandas Development Team, McKinney, W.^35^	https://zenodo.org/records/17992932

**Other**		

Data and Code Availability	This paper	https://github.com/emanuelemassaro/WNV_Europe/

### Experimental model and study participant details

This study did not involve the use of animal models, human participants, plants, microbe strains, or cell lines. All analyses were conducted using the datasets described below and in the manuscript and therefore, no institutional oversight or ethical approval for animal or human subjects was required.

#### Data collection

• MBDs Cases: Human case data for the specified viral diseases—including Chikungunya, Dengue, West Nile virus disease, Yellow Fever, and Zika virus disease—were analyzed at the NUTS-3 level across Europe. Data were collected by each Member State’s Public Health Authority and released by the European Center for Disease Prevention and Control (ECDC) via the European Surveillance System (TESSy).^22^ No further refinement of cases (e.g., clinical manifestation, laboratory confirmation) was performed due to the explorative nature of the study.
• Land Cover Data: Yearly global raster files (300 × 300 m resolution) were sourced from ESA’s Climate Change Initiative (CCI) for 2005–2019.^23^ The temporal dimension was considered, but due to similarity across years, values were averaged.
• Climatic Data: Hourly air temperature (K) and precipitation (m) data were obtained from ERA5 Copernicus.^24^
• Socio-demographic Data: Population, area, population density, and GDP per person at province/NUTS-3 level were retrieved from EUROSTAT^25^ and customized for the study period.

### Method details

#### Variables operationalisation

• Original Variables: Area, population, population density, and GDP were included as reported. Where missing, area and population were used to calculate population density.
• Temporal Averaging: All variables were averaged over the study period (2005–2019).
• Incidence Calculation: Human cases were grouped by province/NUTS-3, and incidence was calculated as cases per population.
• Land Cover Aggregation: Land cover data were rescaled to province/NUTS-3 level and grouped into six macro-categories: forest, shrub, urban, crop, water, and other. The aggregation of ESA CCI Land Cover classes into these categories was undertaken to simplify the analysis and focus on broader land cover trends relevant to our study objectives.^23^ (See Table S5 in Supplementary Information for detailed class definitions and aggregation.)
• Climatic Variables: Temperature and precipitation were averaged by province/NUTS-3 and season (Winter: Jan–Mar, Spring: Apr–Jun, Summer: Jul–Sep, Autumn: Oct–Dec).^24^ Six derived variables were created:
  o Total growth days (avg. temp >14.3°C per season)
  o Maximum growth days (max consecutive days >14.3°C per season)
  o Total wet days (precipitation >0.001 m per season)
  o Total dry days (precipitation ≤0.001 m per season)
  o Maximum wet days (max consecutive days >0.001 m per season)
  o Maximum dry days (max consecutive days ≤0.001 m per season)

### Quantification and statistical analysis

• Descriptive Statistics: Incidence of human MBD cases (2005–2019) and average land cover values were reported, alongside socio-demographic factors and mean seasonal climatic variables.
• Regression Analysis: All dependent and independent variables were standardised (z-scores) as follows:

#### Predictors (X)

All covariates were standardised using classical z-scaling (mean = 0, SD = 1) with the StandardScaler implementation from scikit-learn. This would ensure that all predictors contribute on the same scale and that regression coefficients are comparable across variables. Standardisation was performed globally across all NUTS-3 units, not separately by country or region.

#### Dependent variable (Y)

To avoid mixing the scale of raw incidence with its spatially filtered counterpart, we first computed the spatial lag of incidence:

Wy=Wy

using a row-standardised spatial weights matrix W (DistanceBand, 150 km). Only after constructing the spatial lag did we apply z-standardisation

Wyz=Wy−mean(Wy)std(Wy)

This ensures that the OLS and GWR models operate on the same derived outcome, allowing coefficients to be interpreted as changes in the standardised spatially lagged incidence.

Pearson correlation matrix and the Variance Inflation Factors (VIFs)^36^ were calculated for all standardized covariates to identify and exclude variables with correlation ≥0.8, prioritising known relevant factors (e.g., spring temperature) (see Table S4). As expected, high collinearity was observed among climate-related variables, particularly between seasonal temperature indicators (Mean_T_Spring–Mean_T_Summer, VIF = 144.9–187.9) and degree-day variables (Max_DD_Winter–Max_DD_Autumn, VIF ≈10.7). These patterns reflect the shared climatic gradient across Europe and are ecologically meaningful.

• Final Model Variables:
  o Land cover (forest, shrub, urban, crop, water, other)
  o GDP
  o Population density
  o Mean temperatures (spring, summer)
  o Mean precipitation (winter)
  o Maximum wet days (winter, autumn)
  o Maximum dry days (winter, spring, autumn)
• Spatial Autocorrelation: Moran’s I was calculated to assess spatial autocorrelation (see Table S2).^37^
• Spatial Lag Model: A spatial lag model was implemented to account for spatial dependencies, using a distance-based spatial weights matrix (max distance: 150 km - see Table S1). Let *y*_*i*_represent the value of the dependent variable at location *i*. The spatial lag model introduces a spatially lagged dependent variable, *Wy*, where *W*is the spatial weights matrix. The element *W*_*ij*_of the matrix *W*quantifies the spatial relationship between locations *i*and *j*. To define the spatial weights matrix (*W*), we employed a distance-based approach: neighbors were selected on a metric base, with the dataset first transformed to metric units and a distance matrix developed ranging from 0 to a maximum of 150 km. This distance matrix was used to weight the dependent variable. The independent variables were instead weighted using the bandwidth selected for the geographically weighted regression (GWR) model. We selected an adaptive bandwidth with a bi-square kernel, and validation of this bandwidth was based on the Akaike Information Criterion (AIC). The spatially lagged dependent variable for location *i*is then defined as

$${\left(Wy\right)}_{i}=\sum_{j=1}^{n}W_{ij}y_{j}$$

where *n*is the total number of observations. The adjusted dependent variable $y_{i}^{*}$ incorporates the lag effect and is expressed as

$$y_{i}^{*}={\left(Wy\right)}_{i}$$

A sensitivity analysis was performed by varying the distance-band threshold used to construct the spatial weights matrix from 100 to 200 km in 25 km increments. For each distance, we examined (i) the connectivity of the spatial weights and (ii) the level of spatial autocorrelation in the OLS residuals (see Section SI.1 in SI; Tables S1, S2 and S3). Thresholds below 150 km produced increasingly sparse weight matrices (e.g., 28 units with no neighbors at 100 km; 6 at 125 km), whereas thresholds ≥150 km substantially reduced the number of isolates (4 at 150 km; 3 at 175 km; 2 at 200 km) while avoiding excessively dense neighbor structures. Moran’s I on the OLS residuals decreased monotonically with distance (0.21 at 100 km → 0.09 at 200 km), but remained positive and highly significant for all thresholds tested (*p* ≤ 0.002), indicating persistent spatial autocorrelation in the residuals regardless of the specific choice of d. Based on this analysis, d = 150 km was selected as it minimises the number of spatial isolates and yields a well-connected weights matrix while keeping spatial interactions local. To identify an appropriate threshold distance for the spatial weights matrix, a systematic sensitivity analysis was performed for $d∈ {100, 125, 150, 175, 200 [km]} $. The threshold of 150 km was selected because it reduces spatial isolates to a minimal level (4 units), preserves an interpretable number of neighbors, and ensures GWR residuals lack significant spatial autocorrelation. Multicollinearity was evaluated using Variance Inflation Factors (VIF). High VIFs in seasonal temperatures (Spring and Summer) led us to retain only Mean_T_Spring in the final model to avoid redundancy. Finally, ESA CCI land-use classes were aggregated into broader macro-categories to reduce complexity while retaining ecological relevance (see Table S5 in SI).

• Geographically Weighted Regression (GWR) with Spatial Lag (SL): We employed a Geographically Weighted Regression (GWR) model to account for spatial heterogeneity in the relationship between dependent and independent variables, incorporating the spatially lagged dependent variable ($y_{i}^{*}$) to address spatial autocorrelation.^36^^,^^38^ The GWR-SL model was implemented in two steps: selection of the optimal bandwidth using the Akaike Information Criterion (AIC), and model fitting. GWR is a local linear regression technique that allows regression coefficients to vary spatially, revealing local patterns that may be masked in global models. The GWR-SL model is formulated as

$$y_{i}^{*}=\beta_{0i}+\sum_{k=1}^{p-1}\beta_{ki}x_{ki}+\epsilon_{i}$$

where coefficients are estimated for each location using

$$\beta_{i}={\left(X^{T}W_{i}X\right)}^{-1}X^{T}W_{i}Y$$

with *W*_*i*_as a location-specific diagonal matrix of spatial weights. Weights were assigned using a bisquare kernel function with adaptive bandwidth, determined by minimising the corrected AIC (AICc) via golden-section search (mgwr package). The weight for observation *j*at location *i*is

$$w_{ij}={\left(1-{\left(d_{ij}/b\right)}^{2}\right)}^{2}\text{if}\;d_{ij}\leq b$$

and zero otherwise, where *b*is the distance to the Nth nearest neighbor. The optimal bandwidth was stable across all sensitivity scenarios (77 nearest neighbors), indicating robustness in the spatial scale of local relationships (see Table S3).

• Software: All analyses were conducted in Python (version v3.14.0). Single libraries and functions are included in the key resource tables.
