# Supplementary material for: Spatial role of land cover on West Nile virus disease in Europe
Source: iScience. 2026 Apr 15;29(6):115754. doi: 10.1016/j.isci.2026.115754 (PMC13273564; doi:10.1016/j.isci.2026.115754)
Supplement: Document S1. Tables S1–S5 [file mmc1.pdf]

iScience, Volume *29*

Supplemental information

Spatial role of land cover on West

Nile virus disease in Europe

Nicola Riccetti, Alessandro Cescatti, Juan Carlos Ciscar, Grégoire Dubois, Angela Fanelli, Jordi Figuerola, Dolores Ibarreta, Wojciech Szewczyk, and Emanuele Massaro

Table S 1. Connectivity properties of the spatial weights matrix Note: Thresholds below 150 km leads to sparse weight matrices with many isolates. Distances≥150km produce well-connected systems

d (km)	Mean neighbors	Min	Max	Isolates
100	18.32	0	59	28
125	27.78	0	81	6
150	38.93	0	114	4
175	51.78	0	144	3
200	66.23	0	188	2

Table S 2. Spatial autocorrelation of OLS residuals Note: OLS residuals show persistent positive spatial autocorrelation for all distances, confirming a spatial model is required.

s	Moran's I	p-value
100	0.2103	0.002
125	0.1547	0.001
150	0.1293	0.001
175	0.0999	0.001
200	0.0897	0.002

Table S 3. GWR residual spatial autocorrelation Note: For $d \geq 125km$, Moran's I is statistically insignificant, confirming the model removes spatial autocorrelation.

d (km)	Bandwidth	AICc	Moran's I	p-value
100	77	-14041	0.0251	0.023
125	77	-14041	0.0072	0.167
150	77	-14041	0.0033	0.264
175	77	-14041	0.0027	0.271
200	77	-14041	0.0042	0.183

Table S 4. Variance Inflation Factors (VIFs) for all predictors Note: Predictors with VIF<10 were retained to ensure the model is not unduly affected by multicollinearity.

**Variable**	**VIF**
Mean T Summer	187.94
Mean T Spring	144.86
Max DD Autumn	10.79
Max DD Winter	10.64
Max WD Winter	5.14
Mean P Winter	4.34
Max DD Spring	3.84
Max WD Autumn	3.61
Crop	2.24
Forest	1.89
gdp	1.62
Urban	1.59
Other	1.49
Shrub	1.4
pop_dens	1.33
Water	1.24

Table S 5. Aggregation of ESA CCI classes into macro-categories.

Macro-category	ESA CCI classes aggregated
**Forest**	Tree cover, broadleaved/needleleaved, evergreen/deciduous, closed/open; Mosaic tree and shrub (>50%)
**Shrub**	Mosaic herbaceous cover (>50%); Scrubland; Evergreen/Deciduous shrubland
**Urban**	Urban areas; Bare areas (consolidated and unconsolidated)
**Crop**	Cropland (rain-fed/irrigated); Herbaceous cover; Tree or shrub cover; Mosaic cropland (>50%)
**Water**	Water bodies
**Other**	Grassland; Lichens and mosses; Sparse vegetation/tree/shrub; Flooded vegetation; Permanent snow/ice
